# Severe pre-eclampsia complicated by HELLP syndrome alterations in the structure of the umbilical cord (morphometric and immunohistochemical study)

**DOI:** 10.1080/13102818.2014.991545

**Published:** 2015-01-19

**Authors:** Deniz Balsak, Cihan Togrul, Cenap Ekinci, Yunus Cavus, Ali Emre Tahaoglu, Engin Deveci, Talip Gül, Evren Karaman, Aysun Ekinci, Nafi Sakar

**Affiliations:** ^a^ Department of Obstetrics and Gynecology, Diyarbakır Maternity and Child Health Hospital, Diyarbakır, Turkey; ^b^ Department of Histology and Embryology, Dicle University, School of Medicine, Diyarbakır, Turkey; ^c^ Department of Obstetrics and Gynecology, Memorial Hospital, Diyarbakır, Turkey; ^d^ Department of Obstetrics and Gynecology, Faculty of Medicine, Dicle University, Diyarbakır, Turkey; ^e^ Department of Biochemistry, Faculty of Medicine, Dicle University, Diyarbakır, Turkey; ^f^ Department of Obstetrics and Gynecology, Diyarbakır Education Research Hospital, Diyarbakır, Turkey

**Keywords:** HELLP syndrome, umbilical cord, CD44, MMP9, α-smooth actin

## Abstract

The aim of this study was to evaluate the morphometric and immunohistochemistry in umbilical cords from patients with severe pre-eclampsia with and without haemolysis, elevated liver enzymes and low platelets (HELLP) syndrome. The patient and control groups were similar according to baseline obstetric characteristics. White blood cell count in patients with HELLP syndrome and the control group was significantly increased among patients with HELLP syndrome (*p* < 0.001). Morphometric examination and endothelial core length were significantly different between the groups. In the umbilical cord cross-section of the HELLP group, endothelial cell degeneration in the vessel wall and basement membrane thickening were observed. In the muscle layer of blood vessels, the following disorders were found: increased collagen fibres in the muscle cell, hyperplasia and separation of muscle fibres as well as edema in the intermediate connective tissue. Immunohistochemical analysis showed that endothelial cells, basal membrane and fibroblast cells in the HELLP group expressed high levels of CD44. Vessel wall and amniotic epithelial basement membrane thickening were observed in the HELLP group. Matrix metalloproteinase 9 (MMP9) was expressed. Fibroblast and smooth muscle cells were fusiform and showed a positive reaction to immunohistochemical staining of *α*-actin smooth muscle.

## Introduction

HELLP (haemolysis, elevated liver enzymes and low platelets) syndrome is considered to be a severe form of pre-eclampsia with the features of a thrombotic microangiopathy: consumptive thrombocytopenia with the formation of microvascular platelet thrombi, resulting in microangiopathic haemolytic anaemia and hepatic damage.[1]The presence of HELLP syndrome further increases the risk of maternal and neonatal morbidity and mortality.[2] Maternal vascular endothelial dysfunction with resultant impaired synthesis of vasodilatators or excessive production of vasoconstrictors, as well as an increased sensitivity of the vasculature to endogenous pressor substances may play a central pathogenic role in severe pre-eclampsia.[3] The umbilical cord is covered by an epithelium derived from the enveloping amnion. The network of glycoprotein microfibrils and collagen fibrils in Wharton's jelly has been previously studied.[4] The interlaced collagen fibres and small, woven bundles are arranged to form a continuous soft skeleton that encases the umbilical vessels.[5] CD44 is a receptor for hyaluronic acid (HA), which mediates cell-to-cell and cell-to-matrix interactions through its affinity for HA. Adhesion with HA plays an important role in cell migration, tumour growth and progression.[6] *α*-smooth muscle actin is found in vascular walls, intestinal muscularis mucosae and muscularis propria and in the stroma of various tissues.

The purpose of this study was to examine the changes in the umbilical cord in women diagnosed with HELLP syndrome.

## Subjects and methods

### Subjects

The study protocol was approved by the Medical Committee of Diyarbakir hospital Maternity and Child Health Hospital and informed consent was obtained from all subjects involved in the study. Twenty patients with HELLP syndrome and 20 age-matched healthy pregnant women were enrolled in this study (in total 40 pregnant women). All umbilical cords were provided from the Diyarbakır Maternity and Child Health Hospital (Department of Obstetrics and Gynecology). Umbilical cords of babies born at 35–38 weeks of pregnancy were removed. HELLP syndrome (*n* = 20) and normal umbilical cords (*n* = 20) or a total of 40 units were received. New onset hypertension (systolic blood pressure ≥140 mmHg and/or diastolic blood pressure ≥90 mmHg) and proteinuria (>300 mg in 24 h) were observed in all the patients included in the HELLP syndrome group. HELLP syndrome was defined when three of the following criteria were positive in the absence of other pathologic conditions: lactate dehydrogenase (LDH) >600 U/L, aspartate aminotransferase (AST) ≥ 70 U/L or alanine aminotransferase (ALT) ≥ 70 U/L, platelet count <100,000 cells/mm.[7]

Each umbilical cord was immediately clamped at delivery. In all cases, 10–12 cm-long sections of umbilical cord were cut. The specimens were immersed in 10% buffered formaldehyde. They were dehydrated in a graded ethanol series, cleaned in xylene and embedded in paraffin. Then 4 µm sections were cut and made into slides. These were stained with hematoxylin–eosin and Trichrom Masson.

### Immunohistochemical technique

Formaldehyde-fixed tissue was embedded in paraffin wax for further immunohistochemical examination. Sections were deparaffinized in absolute alcohol. Antigen retrieval process was performed twice in citrate buffer solution (pH:6.0), first for 7 minutes, and second for 5 minutes, boiled in a microwave oven at 700 W. They were allowed to cool to room temperature for 30 minutes and washed twice in distilled water for 5 minutes. Endogenous peroxidase activity was blocked in 0.1% hydrogen peroxide for 20 minutes. Ultra V block (Cat.No:85-9043, Invitrogen, Carlsbad, CA, USA) was applied for 10 minutes prior to the application of primary antibodies (Abcam anti-CD44 (ab6124) antibody 1:150), (mouse monoclonal anti-human alpha-smooth-muscle actin; Santa-Cruz 1:100) and matrix metalloproteinase 9 (MMP9) (Sc-21733 mouse monoclonal IgG) 1:100 overnight. Secondary antibody (Cat.No:85- 9043, Invitrogen, Carlsbad, CA, USA) was applied for 20 minutes. Slides were then exposed to streptavidin–peroxidase for 20 minutes. Chromogen diaminobenzidine (DAB Invitrogen,Carlsbad,CA,USA) was used. Control slides were prepared as mentioned above, but omitting the primary antibodies. After counterstaining with hematoxylin, and washing in tap water for 8 minutes and in distilled water for 10 minutes, the slides were mounted with entellan.

### Statistical analysis

Statistical analyses of the data were conducted by using Statistical Package for Social Sciences (SPSS) version 15.0 (Chicago, IL). Data were presented as mean ± standard deviation. Normality of variance was evaluated with the Kolmogorov–Smirnov test. Variables with non-parametric distribution were compared using the Mann–Whitney U test. A *P* value less than 0.05 was accepted as statistically significant.

## Results and discussion

**Figure 1.**
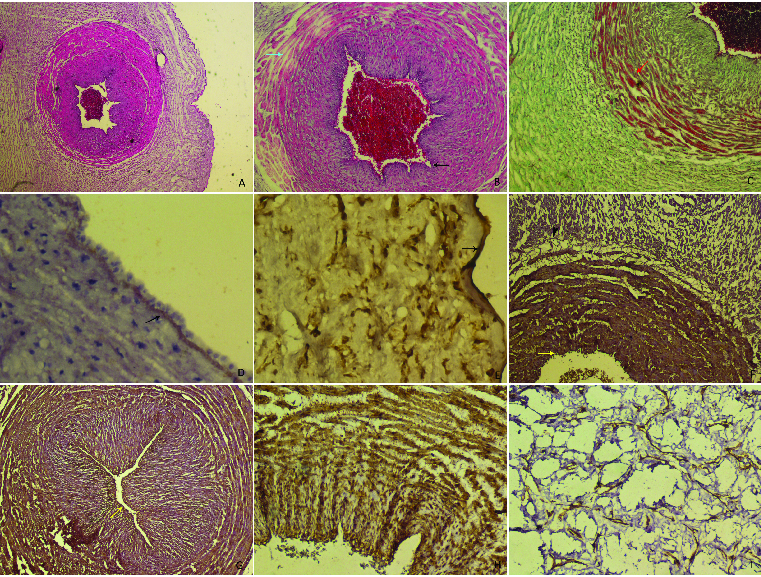
**A**: Control group, appearance normally of umbilical cord, H–E stain Bar 50 µm. **B and C:** HELLP group; degeneration of endothelial cell in the vessel wall (arrows), thickness in basal membrane, edema in the intermediate connective tissue between muscle layers (arrows), H–E stain Bar 100 µm, increase in collagen fibres between smooth muscle layers (arrow), Tichrom-Masson stain Bar 100 µm. **D and E:** Strong expression of MMP9 in basal membrane of vessel wall and amniotic epithelium (arrows). Also positive expression in Wharton's jelly. MMP9 immunohistochemistry staining Bar 100 µm. **F and G:** Positive expression in endothelial cells, basal membrane and fibroblast cells in HELLP group, CD44 immunohistochemistry staining Bar 50 µm. **H and I:** Positive reaction of actin protein in Wharton's jelly spindle-like fibroblast cells and smooth muscle layers. α-smooth muscle actin immunohistochemistry staining Bar 100 µm.

A total of 20 patients with HELLP syndrome were compared to 20 healthy pregnant controls with respect to obstetric characteristics, haematological, biochemical and umbilical cord morphological parameters, as well as indicated by histochemical and immunohistochemical staining. There was no significant difference between the groups according to baseline obstetric characteristics but in the group with HELLP syndrome systolic and diastolic blood pressure, liver function tests and LDH were significantly increased whereas platelet count was significantly lower in HELLP relative to healthy controls (Tables 1 and 2). White blood cell count (WBC) was significantly increased in patients with HELLP syndrome. Morphometric examination, endothelial core length, among all the parameters, were significantly different between the groups (Tables 3 and 4). In the control group, the normal appearance of the blood vessels in the umbilical cord and amniotic membrane. Also, Wharton's Jelly did not show any changes in the fibroblasts in the muscle tissue (Figure 1A). In the umbilical cord cross-section of the HELLP group, endothelial cell degeneration in the vessel wall and basement membrane thickening were observed. In the muscle layer of blood vessels, the following disorders were found: increased collagen fibres in the muscle cell, hyperplasia and separation of muscle fibres as well as edema in the intermediate connective tissue. Wharton's jelly in the HELLP group had wide gaps between collagen fibres compared to the control group (Figure 1(B), 1(C)).

**Table 1.** Obstetric characteristics of the HELLP syndrome and control group.

Characteristics	HELLP group	Control group	*p*
Maternal age (years)	27.4 ± 6.7	28.4± 4.6	0.512
Number of living children	1.4± 2.14	2.14± 1.52	0.058
Gestational age (week)	32.8± 3.34	33.6± 4.1	0.187
Systolic blood pressure (mmHg)	162.3± 19	119.8± 6.6	<0.001
Diastolic blood pressure (mmHg)	105.7± 15	73.4± 5.7	<0.001

Data are presented as mean± standard deviation

**Table 2.** Hematological and biochemical parameters in both groups.

	HELLP group	Control group	*p*
HGB	10.3± 1.6	11.8± 1.18	0.161
WBC	15148± 4178	10810± 2892	<0.001
PLT	93.2± 48.8	252± 64.4	<0.001
ALT	232± 186	17± 7	<0.001
AST	207± 125	23± 9	<0.001
LDH	902± 355	283± 119	<0.001

Data are presented as mean± standard deviation, HGB:Hemoglobin (gram/deciliter), WBC: White blood cell (cells/microliter), PLT: Platelet (cells/microliter), AST: Aspartate aminotransferaseU/L, ALT: Alanine aminotransferaseU/L, LDH: Lactate dehydrogenase (international unit/liter)

**Table 3.** Morphometric parameters in both groups.

	HELLP*n* = 20	Control*n* = 20	*p*
Group	Mean (SD)Median (SEM)Range		Mann–Whitney U Test
Arterial wall thickness	95.3 (8.06) 96.9 (2.54) 82.7–105	71.1 (7.73) 70.4 (2.44) 56.3–81.3	<0.001
Vein wall thickness	78.6 (8.84) 80.9 (2.79) 63.8–88.9	63.0 (10.7) 68.9 (3.39) 45.3–73.3	0.007
Basement membrane thickness	9.07 (2.41) 8.70 (0.76) 6.06-14.9	4.63 (0.76) 4.73 (0.24) 3.57–5.73	<0.001
Amnion cell nucleus length	8.34 (2.06) 8.51 (0.65) 4.25–11.7	4.54 (1.34) 4.38 (0.42) 3.04–7.39	0.001
Endothelial cell nucleus length	7.27 (1.38) 7.67 (0.43) 4.70–8.80	6.16 (1.54) 6.22 (0.48) 3.40–8.57	0.096
Fibroblast cell nucleus length	10.0 (0.77) 9.91 (0.24) 9.02–11.4	6.27 (1.06) 6.34 (0.33) 4.15–7.81	<0.001
Smooth muscle cell nucleus length	12.3 (2.12) 12.6 (0.67) 9.51–15.6	8.62 (1.47) 8.33 (0.46) 6.70–10.7	0.002

**Table 4.** Morphometric graphics.

Note: A – arterial wall thickness, B –vein wall thickness, C – basal membrane thickness, D – amnion epithelial length, E – endothel cell nucleus length, F – fibroblast nucleus length, G – smooth muscle nucleus length.

In the HELLP group, there was a thickening in the vessel wall and the amniotic epithelial basement membrane, immunoreaction for MMP9 was positive (Figure 1 (D), 1(Е)). Immunohistochemical analysis showed that endothelial cells, basal membrane and fibroblast cells in the HELLP group expressed high levels of CD44 (Figure 1 (F), 1(G)). Fibroblast cells were fusiform or spindle-form cells and showed a positive reaction to immunohistochemical staining for *α*-actin smooth muscle (Figure 1(H, 1(I)).

A variant of severe pre-eclampsia, HELLP syndrome is usually considered to be a life-threatening complication of pregnancy. Abnormal alterations in morphology and biochemical properties of the umbilical cord are associated with pathologic processes that occur in pregnancy, such as pre-eclampsia, fetal growth restriction, and diabetes.[8,9] Chronic hypertension is characterized by an increased vascular resistance and modifications in the mechanical properties of blood vessels. [10] The umbilical arterial endothelium is significantly affected in pre-eclampsia patients.[11] Compared to the normal pregnancy, the cord from HELLP syndrome showed different values of the parameters in all components of Wharton's jelly, vein and arteries, and the differences were statistically significant in all cases. In a previous study, it was shown that the wall of the umbilical cord artery is decreased in pre-eclampsia patients.[12] Junek et al. [13] reported increased thickness of the tunica media and intima in the arteries and an increased rate of duplication of the internal elastic lamina in pre-eclamptic cords. In our study, the HELLP group demonstrated increased thickening of the artery walls.

Wharton's jelly is a soft connective tissue and consists mainly of fibroblasts and macrophages, which are embedded in a homogeneous jelly-like intercellular substance.[14] Wharton's jelly acts as a protective barrier on the wall structure of the vein and arteries. In the HELLP group, the disorder of collagen fibres in Wharton's Jelly lead to expansion in space, which might lead to deterioration in the structure of blood vessels.

According to a previous study, the widening of the media, an increased number and thickening of elastic lamella, decreased cellularity and augmented collagen content characterize the morphologic development during this period. In addition, it was reported that the umbilical perfusion decreased in pre-eclampsia.[15]

CD44 angiogenesis, resulting in endothelial cell proliferation, migration and differentiation, may contribute to the synergistic stimulation. Immunohistochemical analysis showed that endothelial cells, basal membrane and fibroblast cells expressed high levels of CD44.The role of CD44 hyaluronate interactions may be important for differentiation of endothelial cells during angiogenesis. MMP secretion by microvascular endothelial cells is an essential first step in the formation of angiogenesis. According to Galewska et al. [16], the umbilical cord plasma of pre-eclamptic subjects contained large amounts of MMP9 in the form of complexes with other plasma components, and zymographic analysis demonstrated increased gelatinolytic activity at a position corresponding to MMP9, compared to control samples. In our study, expression of MMP9 in the basement membrane, Wharton's jelly showed a significant increase in the matrix.

Except the endothelial cells, all cells of the umbilical artery and vein stained strongly for *α*-smooth muscle actin. Fibroblast cells in our study were fusiform or spindle-form and showed a positive reaction to immunohistochemical staining for *α*- smooth muscle actin.

It has been found that in Wharton's jelly of severe pre-eclamptic patients, there is a higher level of collagen and glycosaminoglycans.

## Conclusions

HELLP syndrome induces a decrease in the elastin content accompanied by thickening of the vessel wall in umbilical cord arteries. This change may cause a decrease in the elasticity of Wharton's jelly and, in consequence, may reduce the ability of the umbilical cord vessels to regulate their diameter in response to alterations of the blood pressure during the prenatal period or delivery and may also disturb fetal blood circulation.
